# Castleman disease with synchronous primary rectal and prostate cancers: a case report of successful multimodal treatment and 2-year recurrence-free survival

**DOI:** 10.3389/fonc.2025.1613935

**Published:** 2025-08-13

**Authors:** Linhai Zheng, Mudan Lu, Yiming Liu, Zijian Qiu

**Affiliations:** ^1^ Department of Hernia and Pediatric Surgery in Gastroenterology Center, The Quzhou Affiliated Hospital of Wenzhou Medical University, Quzhou People’s Hospital, Quzhou, China; ^2^ Ward of 5-11-1, The Quzhou Affiliated Hospital of Wenzhou Medical University, Quzhou People’s Hospital, Quzhou, China; ^3^ Department of Pathology, The Quzhou Affiliated Hospital of Wenzhou Medical University, Quzhou People’s Hospital, Quzhou, China; ^4^ Department of Radiation Oncology, The Quzhou Affiliated Hospital of Wenzhou Medical University, Quzhou People’s Hospital, Quzhou, China

**Keywords:** castleman disease, synchronous malignancies, double primary cancers, comprehensive treatment, case report

## Abstract

**Background:**

Castleman disease (CD) is a rare lymphoproliferative disorder associated with immune dysregulation that may increase the risk of malignancy. Synchronous multiple primary cancers are uncommon, and their etiology remains largely unclear. The coexistence of CD with synchronous multiple primary malignancies is exceptionally rare; therefore, the underlying mechanisms and optimal treatment strategies deserve further investigation.

**Case Presentation:**

A 72-year-old male was diagnosed with Castleman disease in the hepatogastric space concomitant with synchronous primary rectal and prostate cancers. The patient underwent resection of the Castleman lesion, followed by four cycles of neoadjuvant FOLFOX chemotherapy for rectal cancer, simultaneous external beam radiotherapy for both rectal and prostate cancers, a Dixon procedure for rectal cancer, and three cycles of adjuvant FOLFOX chemotherapy. During a 2-year follow-up period, serial measurements of total and free prostate-specific antigen (PSA) remained within normal limits, and contrast-enhanced computed tomography (CT) revealed no evidence of recurrence or metastasis. The patient remains clinically stable.

**Conclusion:**

Three tumors in the same patient are extremely rare. The comprehensive treatment plan, including surgery, neoadjuvant chemoradiotherapy, and endocrine therapy may serve as a useful clinical reference for managing similar cases.

## 1 Introduction

Castleman disease (CD), also known as angiofollicular lymph node hyperplasia or giant lymph node hyperplasia, was first defined and named by Castleman in 1956 (1). It is an uncommon, borderline lymphoproliferative disorder typically characterized by painless lymphadenopathy. CD is classified clinically as either unicentric (UCD) or multicentric (MCD) and histologically into hyaline vascular, plasma cell, or mixed subtypes. UCD most often involves lymph nodes in the mediastinum, hilum, or neck, although lesions may also occur in the abdominal or retroperitoneal regions (2). In contrast, MCD presents with diffuse lymphadenopathy, frequently accompanied by systemic symptoms such as fever, anemia, and weight loss. Given its association with immune dysregulation, CD may predispose patients to malignancies, particularly lymphoma and Kaposi’s sarcoma (3, 4).

Double primary cancers represent the most common form of multiple primary malignant tumors, with synchronous cases being significantly less common than metachronous ones (5). Although the underlying causes of multiple primary cancers have not been fully elucidated, genetic predisposition, gene mutations, lifestyle factors, and environmental influences are believed to play a role (6). Currently, there is no consensus on the treatment of multiple primary malignancies, making the identification of an optimal therapeutic strategy a clinical challenge.

This report describes an extremely rare case of Castleman disease in the hepatogastric space concurrent with synchronous primary rectal and prostate cancers. We provide a detailed account of the comprehensive treatment strategy employed and the clinical insights gained through the management of this patient.

## 2 Case presentation

A 72-year-old Chinese male presented on March 20, 2023, with a history of an intermittently palpable right inguinal mass noted for over 60 years. His medical history was significant for untreated hypertension for one year. Apart from the reducible inguinal hernia, the patient reported no additional symptoms.

A pre-admission contrast-enhanced whole-abdominal CT scan performed on March 17, 2023, identified a nodular lesion in the hepatogastric space measuring approximately 4.2 × 2.8 cm with a CT attenuation value of around 41 HU. The lesion demonstrated marked enhancement in the arterial phase and persistent enhancement during the venous phase, raising the possibility of a gastrointestinal stromal tumor. Additionally, a mildly enlarged lymph node was observed in the hepatogastric space, while no significant retroperitoneal lymphadenopathy was noted. Tumor markers revealed a total PSA (T-PSA) level of 17.47 ng/mL (reference range,0–4 ng/mL) and a free PSA (F-PSA) level of 1.39 ng/mL (reference range,0–1 ng/mL).

A colonoscopy performed on March 21, 2023, revealed a rectal mass and multiple colonic polyps. The pathological examination of the rectal biopsy confirmed adenocarcinoma with the following immunohistochemical profile: CK7 (–), CK20 (+), Her-2 (0), Ki-67 (approximately 80%+), CDX-2 (+), PMS-2 (+), MSH-6 (+), MLH-1 (+), MSH-2 (+), and MUC2 (–). A prostate MRI on March 21, 2023, demonstrated an abnormal enhancing nodule in the left peripheral zone of the prostate, suggesting a neoplasm, along with evidence of a mass in the lower rectum. A rectal MRI on March 23, 2023, further characterized the rectal lesion as follows: 1.The tumor’s inferior margin was 5.7 cm from the anal verge, with a lesion length of approximately 2.3 cm, involving roughly one-quarter of the rectal circumference; 2.Tumor invasion depth was classified as T3a; 3.Regional lymph nodes were positive (N1); 4.The circumferential resection margin (CRM) was negative; 5.There was no evidence of extramural vascular invasion; 6.An abnormal signal was noted in the left peripheral zone of the prostate.

**Figure 1:**
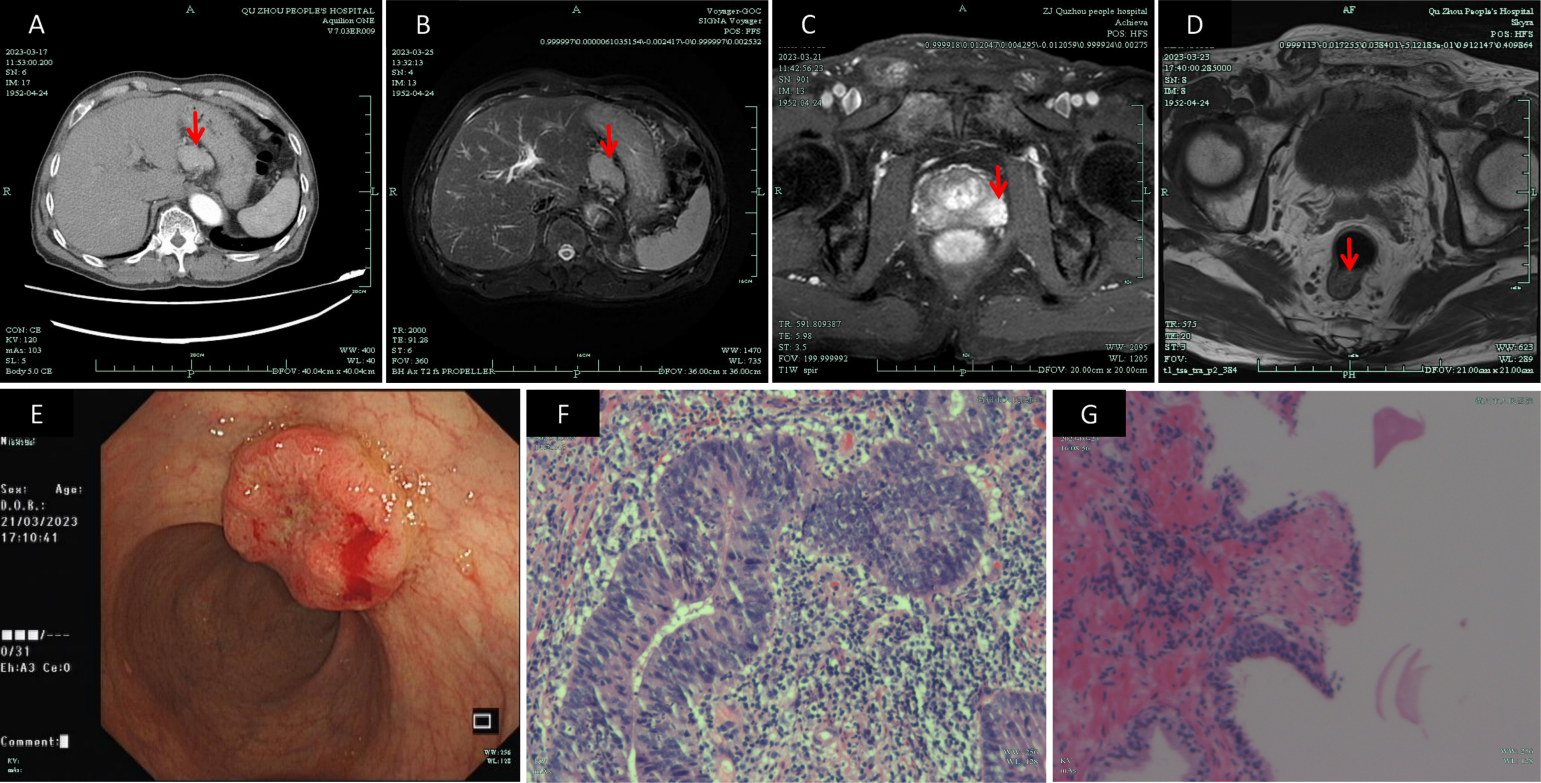
Preoperative imaging studies: **(A)** CT image of the mass in the hepatogastric space (March 17, 2023); **(B)** MR image of the hepatogastric lesion (March 25, 2023); **(C)** MR image depicting an abnormal signal in the left peripheral zone of the prostate and a lesion in the lower rectum (March 21, 2023); **(D)** MR image used for rectal cancer staging (March 23, 2023); **(E)** Colonoscopic image of the rectal mass (March 21, 2023); **(F)** Pathological image from colonoscopic biopsy confirming rectal adenocarcinoma (March 24, 2023)(×400); **(G)** Pathological image from transperineal prostate biopsy confirming prostate adenocarcinoma (March 24, 2023)(×100). CT, Computed Tomography; MR, Magnetic Resonance.

On March 23, 2023, an ultrasound-guided transperineal needle prostate biopsy was performed. The results showed adenocarcinoma in the left prostate lobe with a Gleason score of 5 + 4 = 9 (WHO/ISUP grade group 5) [immunohistochemistry: P63 (–), CK-H (–), P504S (+)], and focal high-grade prostatic intraepithelial neoplasia with a small focus of adenocarcinoma in the right lobe [immunohistochemistry: CK-H (focally negative), P504S (partially positive), P63 (focally negative)]. An upper abdominal MRI performed on March 25, 2023, confirmed the presence of a nodular lesion in the hepatogastric space, favoring a gastrointestinal stromal tumor, although its close proximity to the liver precluded complete exclusion of a hepatic tumor. Mild enlargement of lymph nodes in the hepatogastric space was also noted. These imaging and pathological findings are depicted in **Figure 1**.

Following multidisciplinary team discussion, the preoperative diagnosis was established as a hepatogastric space mass, rectal cancer, and prostate cancer. On March 29, 2023, the patient underwent a laparoscopic resection of the hepatogastric space mass. Intraoperatively, a solitary mass measuring approximately 6 × 5 cm was identified behind the hepatogastric ligament, exhibiting prominent vascular proliferation but no evidence of local invasion. An intraoperative frozen section was consistent with lymphoid hyperplasia suggestive of Castleman disease. Final pathological examination, including hematoxylin and eosin (HE) staining and immunohistochemistry, confirmed the diagnosis of Castleman disease (hyaline vascular type) with the following profile: CD138 (–), Mum-1 (plasma cell +), Cyclin D1 (–), CD10 (–), CD23 (FDC +), CD21 (FDC +), Bcl-6 (germinal center +), BCL-2 (+), Ki-67 (approximately 10%+), CD5 (T-cell +), CD3 (T-cell +), CD20 (+), CK (–), PAX-5 (B-cell +), CD30 (–), and SOX11 (–). Plasma IL-6 levels were measured at postoperative day 11 and 1 year, with values of 13.8 pg/mL and 12.36 pg/mL(reference range,0-5.30 pg/mL), respectively.

**Figure 2:**
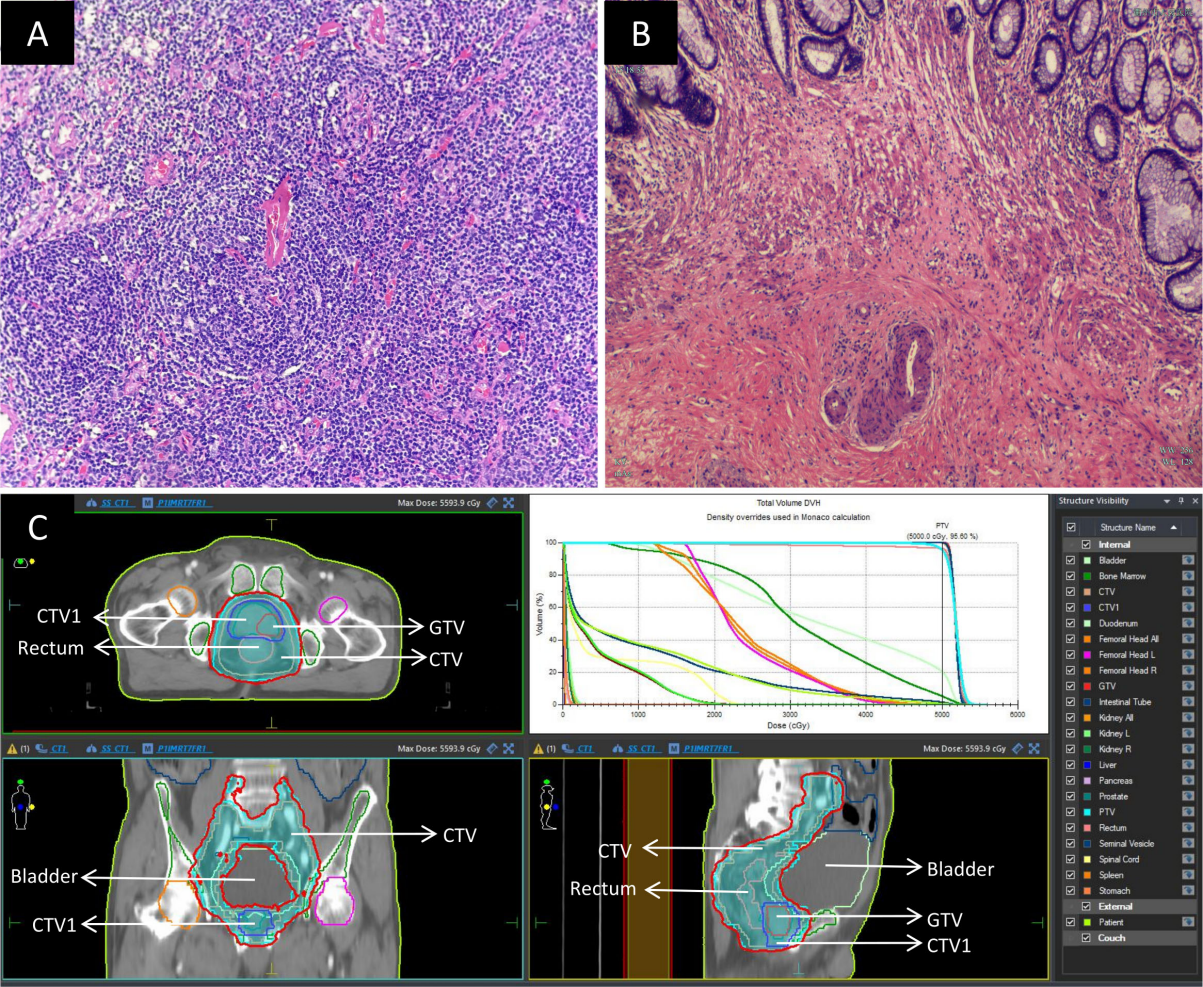
Postoperative pathology and radiotherapy images: **(A)** Histopathological image of the Castleman disease specimen(×200); **(B)** Post-chemoradiotherapy histopathological image of the rectal cancer specimen(×200); **(C)** Images depicting the process of simultaneous radiotherapy to the rectum and prostate. The delineated area of GTV is a prostate tumor. The delineated area of CTV1 is the entire prostate. The delineated area of CTV include rectal and prostate tumors and the pelvic lymphatic drainage areas. To reduce irradiation errors, CTV was expanded outward by 0.5cm to obtain PTV. The prescribed dose of PTV is 50Gy/25 F. The area within the red line represents the actual irradiation of 50Gy after the completion of the radiotherapy plan. GTV, Gross Tumor Volume; CTV, Clinical Target Volume; PTV, Planning Target Volume.

The patient was also diagnosed with rectal cancer (cT3aN1M0, stage IIIb) and prostate cancer (cT2cN0M0, stage II). Given that the rectal tumor was located 5 cm from the anal verge, four cycles of neoadjuvant FOLFOX chemotherapy (comprising oxaliplatin 100 mg, calcium folinate 600 mg, and 4100 mg fluorouracil, administered biweekly) were initiated on April 21, 2023. Simultaneously, starting on April 26, 2023, the patient received external beam radiotherapy to both rectal and prostate cancers (a prescribed dose of 50 Gy in 25 fractions using 6 MV X-ray intensity-modulated radiotherapy, delivered once daily, Monday through Friday, over 5 weeks). Post-radiotherapy, the patient intermittently underwent endocrine therapy with Goserelin (3.6 mg). On August 23, 2023, a laparoscopic radical resection (Dixon procedure) was performed for the rectal cancer. The postoperative pathology report indicated:1. In the rectal specimen, chronic mucosal inflammation with interstitial collagenization was observed, with focal residual mucinous carcinoma cells present; tumor regression was graded as TRG 1; there was no evidence of perineural or vascular invasion, and the proximal, distal, and circumferential margins were negative; 2.Thirteen pericolic lymph nodes were free of metastatic involvement. Immunohistochemical analysis revealed Ki-67 (approximately 40%+), PMS-2 (+), MLH-1 (+), MSH-6 (+), MSH-2 (+), and CK (+).These findings are illustrated in **Figure 2**.

**Figure 3:**
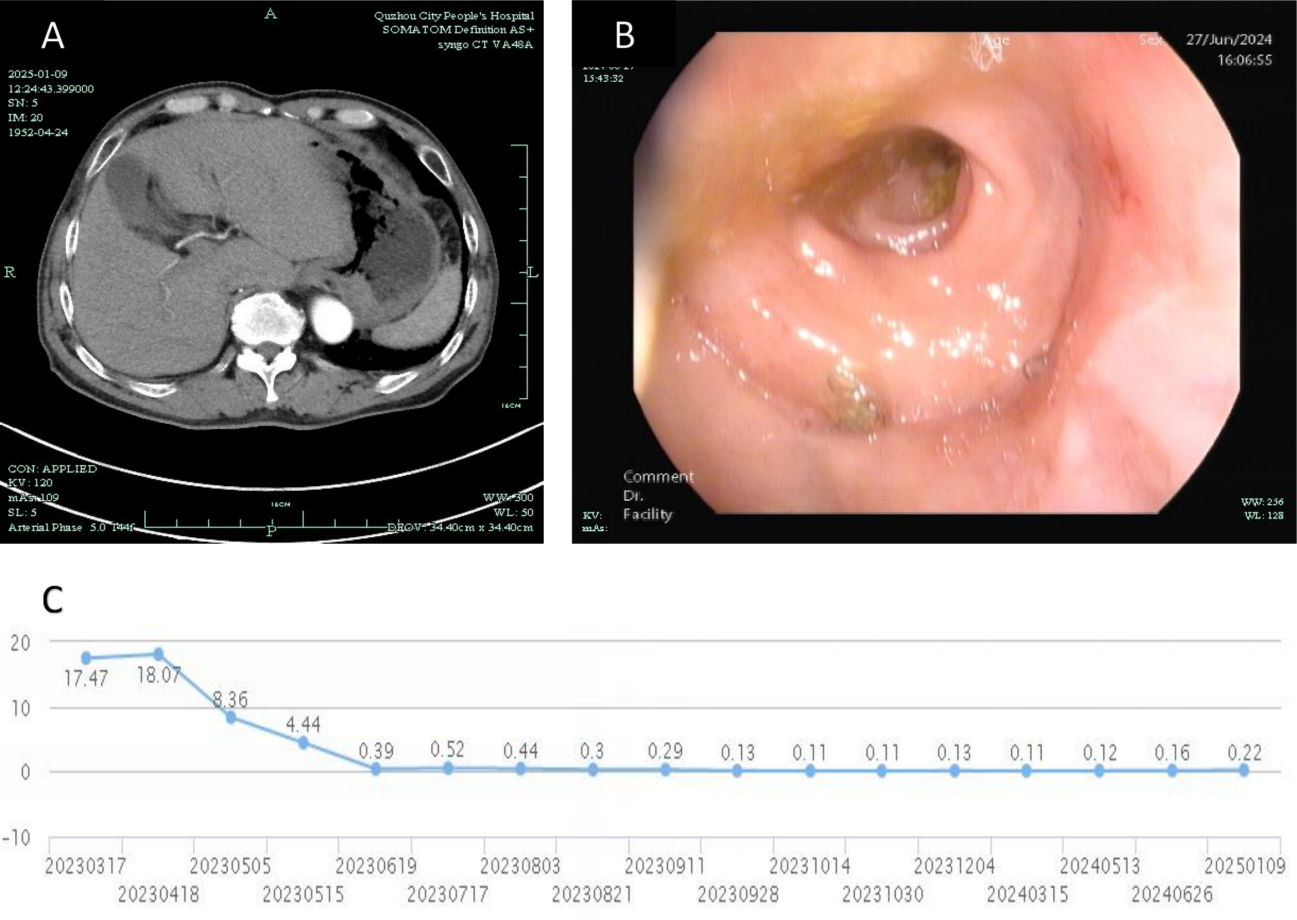
Post-treatment follow-up: **(A)** CT image from January 9, 2025, showing no recurrence in the hepatogastric space; **(B)** Colonoscopic image from June 27, 2024, confirming the absence of recurrence in the colorectum; **(C)** Graph illustrating the trend of total PSA levels over the past 2 years (X-axis ticks represent PSA monitoring timepoints corresponding to actual clinical follow-up dates).

**Figure 4:**
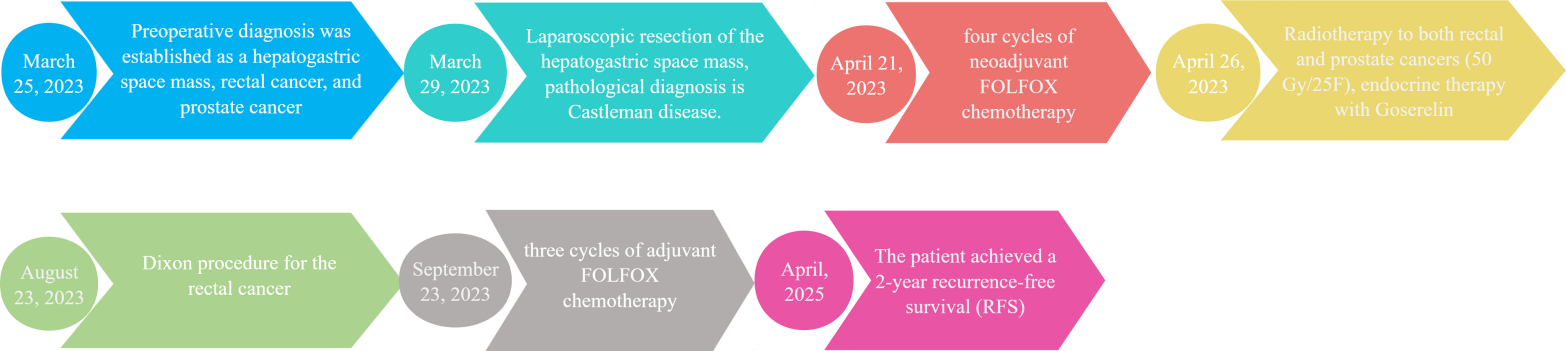
A comprehensive timeline of the treatment.

The patient subsequently received three cycles of adjuvant FOLFOX chemotherapy. Follow-up evaluations, including abdominal CT scans and colonoscopy demonstrated no tumor recurrence, and PSA levels normalized. At the time of reporting, the patient achieved a 2-year recurrence-free survival (RFS), as depicted in **Figure 3**. A comprehensive timeline of the treatment is presented in **Figure 4**.

## 3 Discussion

Castleman disease is characterized by abnormal proliferation of lymphoid tissue with distinct alterations in lymph node architecture. Its clinical presentation and prognosis can vary widely depending on whether the disease is unicentric or multicentric. Epidemiologically, while the incidence of MCD is higher in males, UCD does not exhibit a gender predilection (7). UCD typically manifests as a solitary nodal mass in the neck or abdomen and is often incidentally detected through imaging. For instance, a study in China reported that the most frequently affected nodal regions in UCD were the abdomen (35.7%), the neck (25.7%), and the chest (23.1%) (8). Compared with MCD, UCD generally has a better prognosis, with surgical resection representing the gold standard for treatment (7, 9). In our patient, the abdominal mass was incidentally detected during a preoperative evaluation for an inguinal hernia, and the imaging and pathological findings confirmed UCD. The complete surgical resection has resulted in a favorable outcome, with no recurrence noted on follow-up CT.

Current studies suggest that interleukin-6 (IL-6) plays a central role in the pathogenesis and clinical manifestations of Castleman disease by mediating inflammation, immune response, and hematopoiesis (10). IL-6 is also a critical cytokine in the tumor microenvironment, promoting tumorigenesis through regulation of various signaling pathways involved in apoptosis, proliferation, angiogenesis, invasion, and metastasis (11). In this case, plasma IL-6 levels were measured on postoperative day 11 and at 1 year postoperatively, yielding values of 13.8 pg/mL and 12.36 pg/mL (reference range,0-5.30 pg/mL), respectively. Although these elevated levels suggest that IL-6 may contribute to the disease process, the absence of preoperative IL-6 measurements limits our ability to determine the impact of surgical resection on IL-6 expression.

Preoperative diagnosis of Castleman disease remains challenging (12) and often confused with malignant tumors (13, 14), as unicentric CD lacks specific radiological findings and typically presents as an isolated mass on radiological images. In this instance, the Castleman lesion in the hepatogastric space mimicked a gastrointestinal stromal tumor, resulting in an unexpected diagnostic dilemma. Fortunately, the treatment strategy was not compromised, and the surgical outcome was favorable. Therefore, some scholars advocate obtaining pathological specimens through endoscopic ultrasound-guided biopsy preoperatively to establish a definitive diagnosis (15). Castleman disease has been associated with other neoplasms, including Kaposi’s sarcoma (16), non-Hodgkin lymphoma (17), Hodgkin lymphoma (18), and POEMS syndrome (19, 20). However, its occurrence alongside solid tumors is rare, making the present case of synchronous primary rectal and prostate cancers particularly noteworthy. One limitation of this report is the inability to perform genetic testing due to economic constraints, which would have provided insight into potential molecular alterations.

The incidence of multiple primary malignant tumors is reported to range from 0.73% to 11.70% (21), and these can be classified as synchronous or metachronous. Studies have consistently shown that synchronous tumors occur less frequently than metachronous ones (22). In this case, the incidental diagnosis of synchronous primary rectal cancer and prostate cancer falls within the definition of synchronous multiple primary malignancies.

The rectal cancer in this patient was locally advanced and located near the anal verge, necessitating a standard total neoadjuvant treatment regimen. Colonoscopic biopsy and postoperative pathological immunohistochemistry (IHC) analyses for mismatch repair (MMR) protein expression both indicated proficient mismatch repair (pMMR) status, suggesting limited benefit from immunotherapy. Given the anatomical proximity of the rectum and prostate, simultaneous external beam radiotherapy was administered to both sites, with a total dose of 50 Gy. It is important to note that the patient’s prostate cancer was classified as high-risk (Gleason score 9), for which current guidelines recommend a radical radiotherapy dose of 75–80 Gy combined with endocrine therapy. In our case, the applied radiation dose for the prostate did not reach this radical level, and the patient’s adherence to endocrine therapy was suboptimal. Although PSA levels normalized post-treatment and no recurrence has been detected on imaging, continued monitoring for biochemical or clinical recurrence is warranted. Simultaneous external beam radiotherapy is a feasible option for treating synchronous rectal and prostate cancers (23); however, higher radiation doses to the prostate may lead to significant late complications. Our approach of delivering 50 Gy to the prostate followed by consolidative endocrine therapy appears effective, as evidenced by the patient’s 2-year RFS and absence of serious late complications. Further clinical studies with larger cohorts are needed to optimize management strategies for patients with such complex presentations while considering individualized treatment protocols.

## 4 Conclusion

This report describes an exceedingly rare case of Castleman disease coexisting with synchronous primary rectal and prostate cancers, a clinical scenario for which standard treatment guidelines are not yet established. An individualized, comprehensive treatment plan was employed, including surgical resection of the unicentric Castleman disease, neoadjuvant chemoradiotherapy for locally advanced rectal cancer, simultaneous radiotherapy to both rectal and prostate lesions, and sequential endocrine therapy for prostate cancer. The patient has achieved a 2-year recurrence-free survival, and our experience may provide a valuable reference for managing similar cases.

## 5 Patient perspective

The patient expressed satisfaction with both the therapeutic efficacy and the entire diagnostic-treatment process.

## Data Availability

The original contributions presented in the study are included in the article/supplementary material. Further inquiries can be directed to the corresponding author.
